# LSD1 Activates PI3K/AKT Signaling Through Regulating p85 Expression in Prostate Cancer Cells

**DOI:** 10.3389/fonc.2019.00721

**Published:** 2019-08-02

**Authors:** Zifeng Wang, Shuai Gao, Dong Han, Wanting Han, Muqing Li, Changmeng Cai

**Affiliations:** Center for Personalized Cancer Therapy, University of Massachusetts Boston, Boston, MA, United States

**Keywords:** KDM1A, LSD1, PI3K, AKT, p85, PI3K regulatory subunits, prostate cancer, androgen-deprivation therapy

## Abstract

Lysine specific demethylase 1 (LSD1) functions as a transcriptional repressor through demethylating active histone marks such as mono- or di-methylated histone 3 lysine 4 (H3K4) and interacting with histone deacetylases. However, LSD1 can also act as an activator through demethylating repressive histone marks and possibly non-histone proteins. In prostate cancer (PCa) cells, LSD1 mediates the transcriptional activity of androgen receptor (AR), a ligand dependent nuclear transcription factor that drives PCa initiation and progression to the castration-resistant prostate cancer (CRPC). However, it is unclear whether LSD1 also regulates other growth promoting pathways independent of AR signaling in PCa cells. In this study, we show that LSD1 can activate PI3K/AKT pathways in absence of androgen stimulation, and we further demonstrate that LSD1 transcriptionally regulates the expression of PI3K regulatory subunit, p85, possibly through epigenetic reprogramming of enhancer landscape in PCa cells. Our study suggests that LSD1 has dual functions in promoting PCa development, that it enhances AR signaling through its coactivator function, and that it activates PI3K/AKT signaling through increasing p85 gene expression.

## Introduction

Lysine specific demethylase 1 (LSD1/KDM1A), a specific demethylase of mono- or di-methylated histone lysine 4 (H3K4me1,2, enhancer-associated histone marks), was first identified as a component of REST repressor complex through interaction with CoREST and histone deacetylases 1, 2 (HDAC1,2) (1, 2). LSD1 is also found in Mi-2/nucleosome remodeling and deacetylase (NuRD) repressor complex with interaction with MTA proteins (3, 4). While LSD1 is well-known for its transcription repressor activity, it also activates gene transcription through demethylating repressive histone marks, such as methylated histone 3 lysine 9 (H3K9me1,2), and other non-histone proteins (5–8). In prostate cancer (PCa) cells, LSD1 functions as a major androgen receptor (AR) coactivator (5, 9). This activity was thought to be attributed to androgen-induced phosphorylation of histone 3 threonine 6 and 11 (H3T6/T11ph), which lead to the switch of LSD1 substrate from H3K4me1,2 to H3K9me1,2 (5, 10–12). However, our recent study indicates that the H3K4 demethylase activity of LSD1 persists at AR-mediated enhancers marked with H3T6ph, suggesting additional mechanism(s) mediating its coactivator activity of AR (9). Indeed, we reported that LSD1 interacts and colocalizes with FOXA1, a pioneer factor of AR, at AR-mediated enhancers, and that this interaction may facilitate AR transcription activity (9). Nonetheless, since AR signaling is critical to PCa development and progression to the lethal stage of castration-resistant PCa (CRPC) (13), studies from us and others highly suggest that targeting LSD1 may be a potential treatment strategy for PCa and particularly CRPC, where AR signaling is commonly restored. However, whether LSD1 regulates other major tumor-promoting pathways in PCa cells remains to be determined.

From the RNA-seq analyses of LSD1 inhibitor treated PCa cells, we found that LSD1-activated genes were enriched for PI3K/AKT pathway (14) in absence of androgen stimulation and we further confirmed that LSD1 inhibition significantly decreased AKT phosphorylation independent of DHT treatment. Through functional annotation analyses and subsequent validations, we identified the regulatory subunit of PI3K, p85α (and possibly its isoform p85β), as a critical transcriptional target of LSD1 that mediates its effect on PI3K/AKT pathway activation. Based on these findings, it is plausible that the effectiveness of LSD1 inhibitor treatment in CRPC may be due to inhibition of both AR signaling and PI3K/AKT signaling pathways. As LSD1 inhibitors are currently being tested in clinical trials of leukemia and small cell lung cancer, our studies can be rapidly translated into clinical trials of CRPC.

## Materials and Methods

### Cell Lines and Cell Culture

The LNCaP and CWR22-RV1 cell lines were recently authenticated using short tandem repeat (STR) profiling by DDC Medical (Fairfield). Both cell lines were cultured in RPMI with 10% FBS (fetal bovine serum). For androgen stimulation assays, cells were grown to 50–60% confluence in medium containing 5% charcoal stripped serum (CSS) for 3 days (d) and then treated with DHT or inhibitors for 24 h.

### Chromatin Immunoprecipitation (ChIP)

For preparation of ChIP, dispensed cells were formalin fixed, lysed, and sonicated to break the chromatin into 500–800 bp fragments, followed by immunoprecipitation. Anti-H3K4me2 (Milipore) and anti-V5 (Sigma) are used for immunoprecipitation. The qPCR analysis was carried out using the SYBR Green method. The primers are listed as following: *PIK3R1-*enh: forward, 5′-GTGGAAGAACAGCTTTGGGG-3′, reverse, 5′-TCAAGGCAACTTACTTTGCAGG-3′. *PIK3R1-*LBS: forward, 5′- TTGTTGATTTCCCCACCCCTC-3′, reverse, 5′- TCCCAAGCTGGGCTCTATTTG -3′.

### RT-PCR and Immunoblotting

RNA was extracted with TRIzol Reagent (Invitrogen) following manufacturer's protocol. The expression of genes was measured using real-time RT-PCR analyses with Taqman one-step RT-PCR reagents (Thermo Fisher Scientific) and results were normalized to co-amplified GAPDH. The primer and probe set for the following genes: *FKBP5 (Hs01561006_m1), PIK3R1 (Hs00933163_m1), PIK3R2 (Hs00178181_m1)*, and *GAPDH (4310884E)* were purchased as inventoried mix (Applied Biosystems at Thermo Fisher). For immunoblotting, anti-AKT (Cell Signaling), anti-phosohoylated-473-AKT(Cell Signaling), anti-p85α (R&D), anti-p85β (R&D), anti-H3K4me2 (Milipore), anti-LSD1 (Abcam), anti-V5 (Sigma), anti-HDAC1 (Abcam), anti-GAPDH (Abcam), or anti-β-Tubulin (Abcam) antibodies were used. The inhibitors used are GSK2879552 (Selleck), ORY-1001 (Selleck), S2101 (Calbiochem), tranylcypromine (Calbiochem), and BKM120 (Selleck). Immunoblotting results shown are representative of at least 3 independent experiments.

### Generation of LSD1 Knockout Cell Lines

The effective guided RNA to target LSD1: forward, 5′-CACCGGGGGCCTGGCGGAACCGCCG-3′, reverse, 5′-AAACCGGCGGTTCCGCCAGGCCCCC-3′. The sgRNAs sequences were inserted into lentiGuide-Puro system (Addgene) following the manufacture protocol. Lenti-Cas9-2A-Blast and lentiGuide-Puro vectors were transfected as 1:1 ratio into 22Rv1 cells by Lipofectamine 2000 (Thermo fisher) for 24 h. Cells were then co-treated by blasticidin (1 ug/ml) and puromycin (5 ug/ml) to select single clones. LSD1 knockout in the selected clones was determined by immunoblotting of LSD1.

### Cell Viability Assay

PCa cells were maintained in RPMI-1640 supplemented with 5% CSS. Cell were plated into 96-well plates at ~5,000–10,000 cells/well. After 24 h, cells were treated with DMSO, ORY-1001, and/or BKM-120 for 3 days. The cell viability was then examined by using CellTiter-Glo luminescent cell viability assay (Promega, USA).

### Xenografts

CWR22-RV1 derived xenograft was established in the flanks of castrated male SCID mice by injecting ~2 million cells mixed with 50% Matrigel. LuCaP35CR xenograft tumors were established in the flanks of castrated male SCID mice by transplantation. Tumor volume was measured by manual caliper. Frozen sections were examined to confirm that the samples used for RNA and protein extraction contain predominantly non-necrotic tumor.

### Statistical Analysis

Data in bar graphs represent mean ± SD of at least 3 biological repeats. Statistical analysis was performed using Student's *t*-test by comparing treatment vs. vehicle control or otherwise as indicated. *p*-value < 0.05 (^*^) was considered to be statistically significant. For animal studies, one-way ANOVA was performed for the tumor volume data measured at the final day of the treatments.

## Results

### LSD1 Inhibitor Treatments Decreased AKT Phosphorylation in PCa Cells

**Figure 1:**
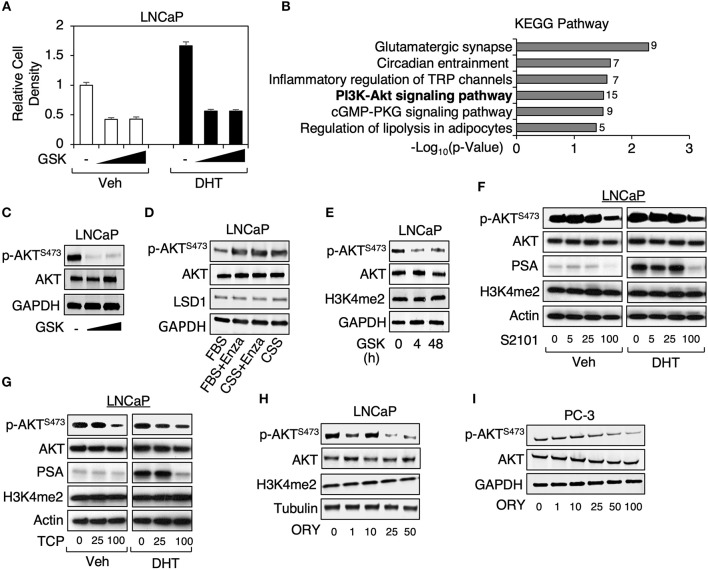
LSD1 inhibitor treatments decreased AKT phosphorylation in PCa cells **(A)** LNCaP cells were maintained in medium containing 0–5 μM GSK2879552 for ~2 weeks (w) and then treated with/out 10 nM DHT for 4 days (d). Cell density was examined under the indicated conditions. **(B)** GSK2879552 (1 μM) downregulated (LSD1-activated) genes (identified from RNA-seq analyses, GSE114268) were analyzed by KEGG pathway annotation. **(C)** Immunoblotting for Ser473-phosphorylated AKT (p-AKT^S473^) and total AKT in LNCaP cells with prolonged treatment of GSK2879552 (0–5 μM). **(D)** Immunoblotting for p-AKT^S473^, total AKT, and LSD1 in LNCaP cells treated with/out 10 μM enzalutamide for 10 days in medium containing full serum (FBS) or hormone-depleted serum (CSS). **(E)** Immunoblotting for indicated proteins in LNCaP cells treated with/out 50 μM GSK2879552 for 0–48 hours (h). **(F–H)** Immunoblotting for indicated proteins in LNCaP cell treated with **(F)** 0–100 μM S2101, **(G)** 0–100 μM TCP, or **(H)** 0–50 μM ORY-1001 in absence or presence of 10 nM DHT for 48 h. **(I)** Immunoblotting for indicated proteins in PC-3 cell treated with 0–100 μM ORY1001 for 48 h.

To identify the additional target pathways of LSD1 independent of androgen treatment, we treated LNCaP cells (an androgen-responsive PCa cells line with *PTEN* loss) under hormone-depleted condition with a clinical tested LSD1 inhibitor, GSK2879552 (Phase I for small cell lung cancer) (15), at lower doses (1 or 5 μM) but for extended period of time (~2 weeks), and then carried out RNA-seq analyses. This treatment resulted in a significant decrease of cell growth regardless of DHT stimulation (Figure 1A). KEGG pathway analysis (provided by DAVID) was performed to identify the enriched functions/pathways in LSD1-activated (LSD1 inhibition-downregulated) and LSD1-repressed (LSD1 inhibition-upregulated) gene subsets. While LSD1-repressed genes were enriched for neuronal and immune responses (data not shown), a consistent finding with its classic function and a recent study (16), LSD1-activated genes were significantly enriched for PI3K/AKT signaling pathway (Figure 1B), which plays a critical role in driving PCa development (14). To determine whether PI3K/AKT pathway is activated by LSD1, we examined Ser473 phosphorylation of AKT, a marker for its full activation (17, 18), in LNCaP cells treated with GSK2879552 (same strategy as in Figure 1A) and our data indicated that LSD1 inhibition markedly decreased AKT phosphorylation (Figure 1C). This result is in sharp contrast to the effects of treating LNCaP cells with an AR antagonist, enzalutamide, or androgen deprivation, which led to increased AKT phosphorylation (Figure 1D) (19).

As we have previously observed that short-term treatment of LSD1 inhibitors required much higher doses (~50–100 μM) to reach the similar effects on cell growth and AR activity in comparison with the prolonged treatment with lower doses (not shown), we next examined whether the high dose treatment can similarly affect AKT phosphorylation in LNCaP cells. As seen in Figure 1E, treating cells with 50 μM GSK2879552 caused rapid inhibition of AKT phosphorylation (4 and 48 h). We then determined whether other LSD1 inhibitors can result in the similar effect on PI3K/AKT signaling. As seen in Figures 1F,G, treatments of two structurally related LSD1 inhibitors, TCP (tranylcypromine) and S2101 (20), resulted in the similar inhibitory effect on AKT phosphorylation at ~100 μM, which also suppressed DHT-induced PSA expression (a classic target of AR). Moreover, we have also examined the effect of another clinical tested LSD1 inhibitor, ORY-1001 (Phase II for AML) (21), on AKT activation. As seen in Figure 1H, ORY-1001 decreased Ser473 phosphorylated AKT at ~25 μM. Furthermore, since LSD1 inhibitor treatment was recently reported to inhibit the growth of AR-negative PC-3 cell-derived xenograft tumors (22), we next examined the effect of LSD1 inhibition on AKT activation in PC-3 cells. As seen in Figure 1I, LSD1 inhibition repressed AKT phosphorylation in PC-3 cells, indicating that this oncogenic activity of LSD1 is distinct from its activity on mediating AR signaling. Overall, these results demonstrate that LSD1 activates PI3K/AKT pathways independent of AR signaling in PCa cells.

### LSD1 Transcriptionally Regulated p85α Expression

**Figure 2:**
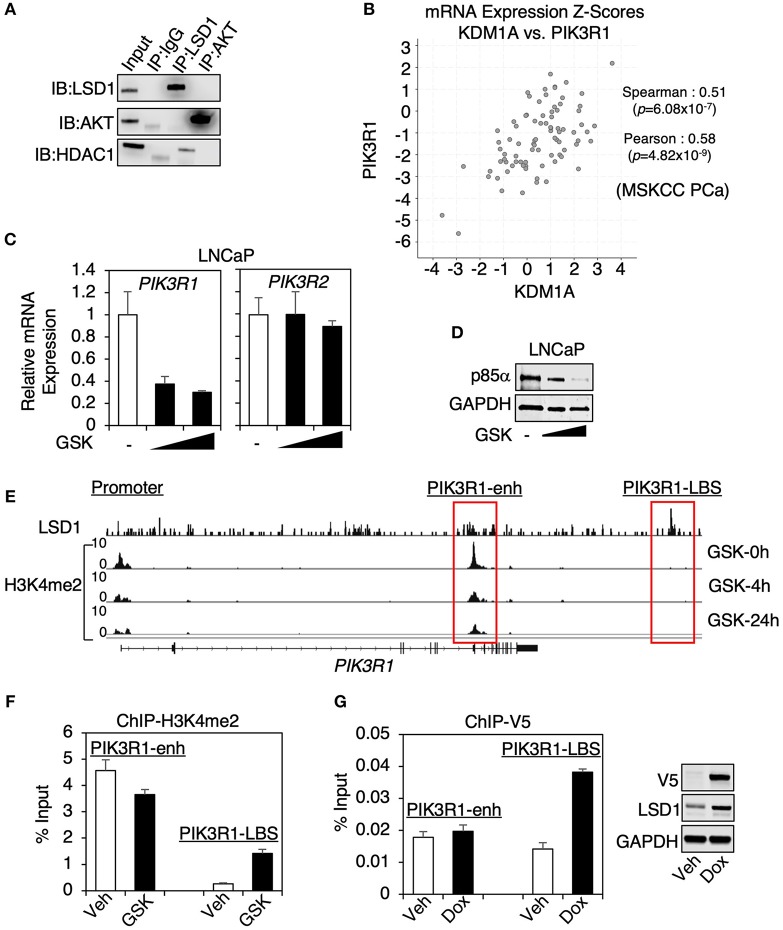
LSD1 transcriptionally regulated p85α expression **(A)** LSD1 or AKT immunoprecipitation was performed in LNCaP cells, followed by immunoblotting for LSD1 and AKT. **(B)** The mRNA expression correlation analyses on KDM1A (LSD1) vs. PIK3R1 (p85α) using MSKCC PCa dataset (*N* = 150) from cBioPortal. The Pearson and Spearman rank correlation coefficient and *p*-value were shown. **(C)** Real-time RT-PCR (qRT-PCR) for PIK3R1 or PIK3R2 in LNCaP cells with prolonged treatment of GSK2879552 (0–5 μM). **(D)** Immunoblotting for p85α in LNCaP cells with prolonged treatment of GSK2879552 (0–5 μM). **(E)** ChIP-seq of H3K4me2 in LNCaP cells treated with 50 μM GSK2879552 for 0–24 h (GSE114268), aligned with public dataset of ChIP-LSD1 (GSE52201) at PIK3R1 gene locus. **(F)** ChIP-qPCR for H3K4me2 binding in LNCaP cells treated with/out 50 μM GSK2879552 for 24 h. **(G)** ChIP-qPCR for V5 (V5-LSD1) in LNCaP-C4-2 cells stably overexpressing tetracycline-regulated V5-tagged LSD1. Doxycycline-induced expression of V5-LSD1 was confirmed using immunoblotting.

Since AKT can be methylated by SETDB1 (although this methylation promotes AKT activity) (23, 24), we first examined whether LSD1 can directly interact with AKT to remove its methylation. However, AKT was not coimmunoprecipitated with LSD1, or vice versa, in LNCaP cells (Figure 2A), indicating that LSD1 is unlikely to demethylate AKT. Therefore, we next hypothesized that LSD1 may transcriptionally regulate an upstream component of PI3K/AKT pathway. Through KEGG analyses, we have identified a subset of LSD1-activated genes that were involved in PI3K/AKT pathways (see Figure 1B). Amongst these genes, *PIK3R1* encodes for regulatory subunit alpha of PI3-Kinase, p85α. In cells, p85 regulatory subunit and p110 catalytic subunit form heterodimer of PI3K, which functions to phosphorylate PI(3,4)P_2_ to PI(3,4,5)P_3_ (25). Although several isoforms of p85, including p85β, are found in PCa cells, p85α is generally the most highly expressed PI3K regulatory subunit (14). Significantly, the expression of *PIK3R1* strongly associated with the expression of *KDMA1* (encoding for LSD1) in MSKCC PCa dataset (using cBioPortal) (26–28) (Figure 2B). We next examined whether p85α expression is regulated by LSD1. As seen in Figure 2C, LSD1 inhibition significantly decreased the mRNA expression of *PIK3R1* but not *PIK3R2* (encoding for p85β). As a result, the protein expression of p85α was markedly reduced by LSD1 inhibitor treatment (Figure 2D). Examining ChIP-seq of H3K4me2 in LNCaP cells, we identified an enhancer site (named *PIK3R1*-enh) located at the gene body of *PIK3R1*, where the level of H3K4me2 was decreased by LSD1 inhibitor treatment (Figure 2E). Surprisingly, using a published ChIP-seq dataset of LSD1, we did not find any LSD1 binding peak at this enhancer. There was only one nearby LSD1 binding site (named *PIK3R1*-LBS) located at the downstream of *PIK3R1* locus, but the level of H3K4me2 is barely detected, indicating that this site is unlikely an active enhancer. Nonetheless, we performed ChIP-qPCR in LNCaP cells to examine the H3K4me2 level at these two sites. As seen in Figure 2F, H3K4me2 was decreased at *PIK3R1*-enh by the LSD1 inhibitor treatment, consistent with the finding from H3K4me2 ChIP-seq. Interestingly, while H3K4me2 was low at *PIK3R1*-LBS, it was increased by LSD1 inhibition, supporting that this site is occupied by active LSD1, which can demethylate H3K4me2. To further determine if LSD1 binds to *PIK3R1*-enh, we generated a stable cell line expressing doxycycline-regulated V5-tagged LSD1 and then performed V5 ChIP. As seen in Figure 2G, LSD1 binding at *PIK3R1*-LBS but not *PIK3R1*-enh was induced by doxycycline treatment, confirming that LSD1 does not directly bind to this enhancer of *PIK3R1*. Overall, these results indicate that LSD1-mediated transcription of *PIK3R1* is possibly due to an indirect activation of a *PIK3R1* enhancer, which may be a consequence of previously reported LSD1-mediated epigenetic reprogramming (29).

### The Combination Treatment of a PI3K Inhibitor With a LSD1 Inhibitor More Effectively Suppressed PCa Cell Proliferation

**Figure 3:**
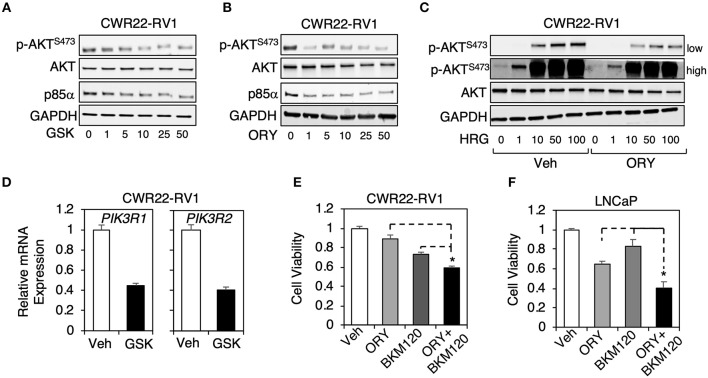
The combination treatment of a PI3-kinase inhibitor with a LSD1 inhibitor more effectively suppressed PCa cell proliferation **(A,B)** Immunoblotting for indicated proteins in CWR22-RV1 cells treated with 0–50 μM **(A)** GSK2879552 or **(B)** ORY1001 for 48 h. **(C)** Immunoblotting for indicated proteins in CWR22-RV1 cells are pre-treated with 10 μM ORY1001 for 48 h and then stimulated by 0–100 ng/ml heregulin-β1 for 10 min. **(D)** qRT-PCR for PIK3R1 or PIK3R2 in CWR22-RV1 cells treated with/out 25 μM GSK2879552. **(E,F)** Luminescence based cell viability assay was performed in **(E)** CWR22-RV1 cells (hormone-depleted serum) or **(F)** LNCaP cells (full serum) treated with vehicle, ORY-1001 (50 μM) alone, BKM120 (1 μM) alone, or the combination of ORY-1001 and BKM120 for 3 days.

We next selected an AR-positive CRPC cell line, CWR22-RV1 cells, to further study the LSD1 function on PI3K/AKT pathway. Interestingly, this cell line appeared to be more sensitive to the LSD1 inhibitors as both p85α expression and AKT phosphorylation were decreased by ~5–10 μM of GSK2879552 or ORY-1001 (Figures 3A,B). The heregulin-induced AKT activation (through activating EGFR and ErbB2 receptors) (30, 31) was also decreased by LSD1 inhibition (Figure 3C). Interestingly, unlike in LNCaP cells where LSD1 inhibition only repressed p85α expression, in CWR22-RV1 cells LSD1 inhibition decreased the mRNA expression of both α and β subunits (Figure 3D).

PI3-kinase inhibitors, such as BKM120, have been tested in clinical trials of metastatic PCa, but failed to demonstrate significant activity in men with CRPC (32). However, our finding that LSD1 regulates p85 expression in PCa cells suggests that the combined treatment of PI3-kinase inhibitor and LSD1 inhibitor may be more effective in inhibiting downstream AKT activation while still maintain suppression effect on AR signaling. To test this hypothesis, we performed cell viability assays in CWR22-RV1 cell line as well as LNCaP cell line treated with BKM120 alone, ORY-1001 alone, or the combination. As seen in Figures 3E,F, the combination treatment was more effective in suppressing cell growth than the single agent treatment, indicating that this treatment strategy may be potentially used in men with CRPC.

### LSD1 Gene Knockout Suppressed PI3K/AKT Signaling in CRPC Cells

**Figure 4:**
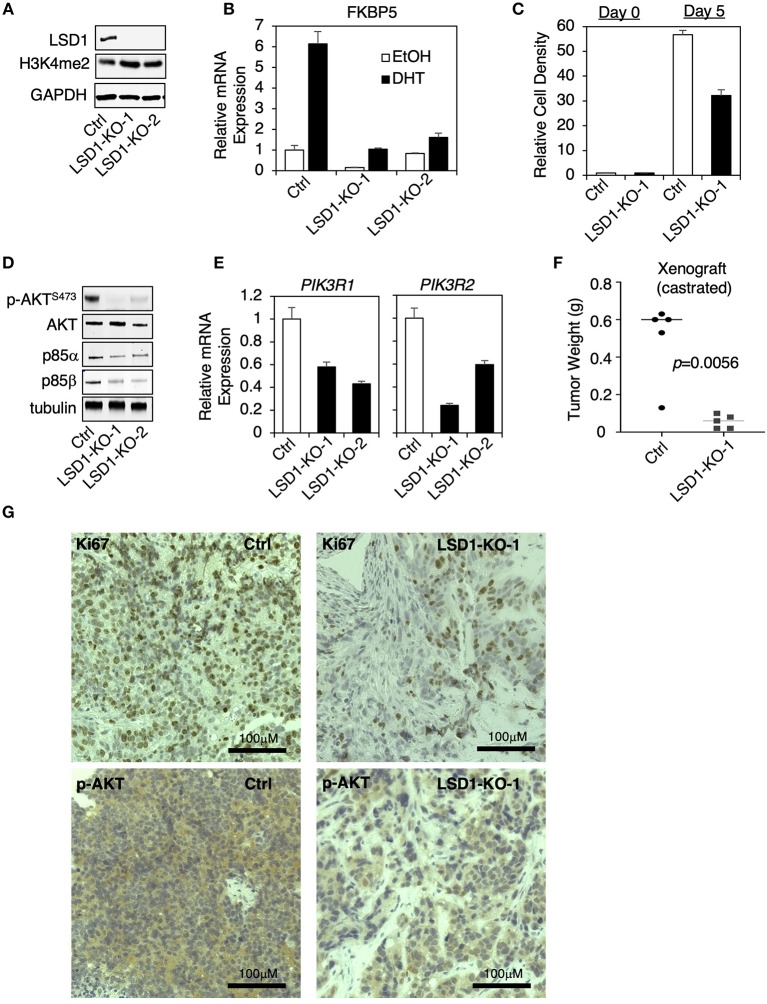
LSD1 knockout suppressed PI3K/AKT signaling in CRPC cells **(A–E)** LSD1 knockout lines (using CRISPR/Cas9 approach) were established in CWR22-RV1 cell. **(A)** LSD1 silencing was confirmed in two knockout clones (LSD1-KO-1 and LSD1-KO-2) compared with parental control line (Ctrl). The following experiments were then performed: **(B)** qRT-PCR for FKBP5 in cells treated with/out 10 nM DHT for 12 h; **(C)** cell viability was measured under the indicated conditions; **(D)** immunoblotting for indicated proteins; and **(E)** qRT-PCR for PIK3R1 and PIK3R2. **(F)** Male SCID mice (*n* = 5) were subcutaneously injected with control line (left flank) and LSD1-KO-1 line (right flank). The development of xenograft tumors was monitored for over 6 weeks and the mice were sacrificed when the tumors at any side reached the size limit. Tumor weight was measured, and tumor biopsies were collected. **(G)** IHC staining of Ki67 and p-AKT^S473^ in tumor samples from control line vs. LSD1-KO-1 line was shown.

Since the above used LSD1 inhibitors may target additional proteins, such as monoamine oxidases, we decided to use CRISPR/CAS9 approach to genetically silence LSD1 expression and then to examine the effect on PI3K/AKT signaling. Two stable clones (LSD1-KO-1 and LSD1-KO-2) were established in CWR22-RV1 cells and selected for the subsequent study (Figure 4A). The AR activity (AR regulation on FKBP5) was markedly impaired in LSD1-KO cells and the cell growth (in absence of androgen stimulation) was significantly reduced (Figures 4B,C). Importantly, AKT phosphorylation and the expression of p85α and p85β were all suppressed by LSD1 knockout (Figures 4D,E), consistent with the inhibitor effects. Furthermore, we also generated the xenograft tumors by injecting the control or LSD1-KO cells in castrated male SCID mice. The tumor growth of LSD1-KO line was much slower and when the tumors in control group exceeded the size limit, we sacrificed the mice and measured the tumor weight. As shown in Figure 4F, the average tumor weight for LSD1-KO line was significantly reduced. This tumor regression effect can be seen by Ki67 immunohistochemistry (IHC) staining (Figure 4G, upper panel). Importantly, the levels of phosphorylated-AKT were significantly reduced in LSD1-KO line (although it was not completely eliminated) (Figure 4G, lower panel), indicating that LSD1 activates AKT pathway *in vivo*. Overall, these results clearly demonstrate that the expression of p85 isoforms and subsequent AKT phosphorylation were specifically regulated by LSD1.

### PI3K/AKT Signaling Was Repressed by LSD1 Inhibition in a Castration-Resistant PDX Model

**Figure 5:**
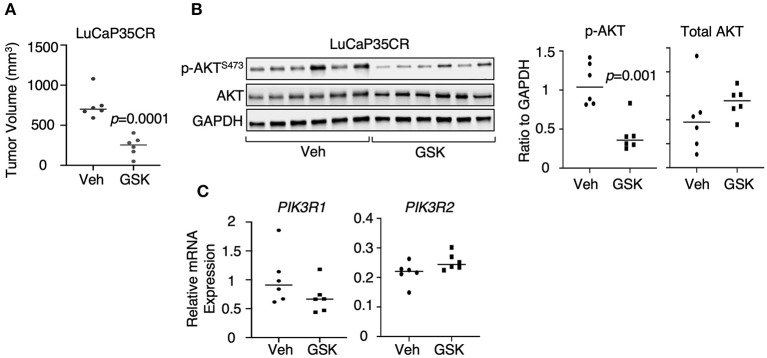
PI3K/AKT signaling was repressed by LSD1 inhibition in a CRPC PDX model **(A)** Castrated SCID male mice bearing LuCaP35CR xenograft tumors (at ~5–7 mm) received daily DMSO (*n* = 6) or GSK2879552 (33 mg/kg) (*n* = 6) via i.p injection for 19 days. The mice were then scarified, and tumor biopsies were collected. The tumor volume at 19 day of treatment was shown. **(B)** p-AKT^S473^ and total AKT were examined by immunoblotting and quantified by ImageJ in tumor tissue samples. **(C)** qRT-PCR for PIK3R1 and PIK3R2 in extracted RNA samples from tumor biopsies.

We next determined whether LSD1 inhibitor treatment can suppress PI3K/AKT signaling in CRPC xenograft models. LuCaP35CR is a previously described patient-derived xenograft (PDX) model that is *TMPRSS2-ERG* positive and *PTEN*-negative, and resistant to androgen deprivation treatment (33, 34). Our study using this model indicates that the tumor growth was inhibited by 3-week GSK2879552 treatment (Figure 5A). Therefore, we examined how this treatment affects PI3K/AKT signaling. As seen in Figure 5B, GSK2879552 treatment significantly decreased AKT phosphorylation, suggesting that the impairment of PI3K/AKT signaling may be one mechanism contributing to the tumor regression effect by LSD1 inhibition. Consistently, *PIK3R1* expression was decreased by the LSD1 inhibitor treatment although it was not statistically significant due to the variations in samples (Figure 5C). Overall, these *in vitro* and *in vivo* studies demonstrated that LSD1 inhibition represses AKT signaling in CRPC cells with different genetic background.

## Discussion

Multiple studies have demonstrated LSD1 as a critical AR coregulator through its activator function, which is independent to its H3K4 demethylase activity (5, 9). This provides a strong molecular basis to treat PCa tumor with LSD1 inhibitors and we are currently testing LSD1 inhibitor treatments in preclinical models of CRPC. However, whether the tumor response to the inhibitors is solely dependent on suppressing AR signaling remains unclear. In this study, we show that LSD1 inhibitors markedly suppressed another major cancer-promoting pathway, PI3K/AKT signaling, in androgen-dependent PCa and CRPC cells, a consistent finding with a study using LSD1 inhibitor S2101 in an ovarian cancer cell line (35). We further demonstrated that this function of LSD1 is, at least in part, due to the transcriptional activation of the regulatory subunit of PI3-kinase, p85α. Interestingly, this activation function of LSD1 appears to be indirect as we observed decreased H3K4me2 by the LSD1 inhibitor treatment at an enhancer of *PIK3R1* (*PIK3R1*-enh), which was not occupied by LSD1 (see Figure 2). One hypothesis is that the distal LSD1 binding site (*PIK3R1*-LBS) can communicate with and subsequently activate *PIK3R1*-enh through chromatin looping and H3K4me-independent activity of LSD1, such as demethylating H3K9 or non-histone proteins or acting as a critical scaffold protein (5, 6, 9, 16, 22, 36). Another hypothesis is that LSD1 can induce an epigenetic reprogramming (29, 37, 38) that would reshape the enhancer landscape in PCa cells. Therefore, this decreased level of H3K4me2 at *PIK3R1*-enh by LSD1 inhibition might be a result of such epigenetic reprogramming. Nonetheless, it remains unclear how LSD1 performs such activation function on the *PIK3R1* enhancer and this unidentified mechanism clearly needs to be determined in the future studies.

Recent studies have indicated that AR signaling and PI3K/AKT signaling are reciprocally regulated by each other in PCa cells (19). Therefore, castration or the standard AR antagonist treatments, such as bicalutamide and enzalutamide (39), commonly result in the unwanted activation of PI3K/AKT signaling (also see Figure 1D), which may lead to anti-apoptotic activity in PCa cells and thus render tumor cells resistant to the therapies. In contrast, the LSD1 inhibitor treatments have dual functions on inhibiting both AR signaling and PI3K/AKT pathways, which provides an advantage to androgen deprivation treatments. In particular, PTEN deficient or PI3K activating mutations are commonly found in primary PCa and CRPC (40, 41) and this subset of PCa have been adapted to the overactivation of PI3K/AKT signaling. Therefore, the LSD1 inhibitor treatments might be more effective to treat this subset of tumors as they target both AR and PI3K/AKT signaling. In addition, since LSD1 regulates p85 gene expression, the combined treatment of PI3K inhibitors which target PI3K enzymatic activity and LSD1 inhibitors may achieve synergistic effect in treating PCa patients and such treatments need to be further tested in the pre-clinical animal models of CRPC. Overall, this study provides novel insights on identifying the downstream effectors of LSD1 in PCa cells and the study will have a strong translational impact as it indicates that the LSD1 inhibitor treatment may be effective in delaying the progression of CRPC as it targets both AR signaling and PI3K/AKT signaling pathways.

## Data Availability

The RNA-seq data used for this study can be found in GSE114268.

## Author Contributions

CC, SG, and ZW designed the study and wrote the manuscript. ZW, SG, DH, ML, and WH performed experiments and analyzed the results. All authors discussed the results and commented on the manuscript.

### Conflict of Interest Statement

The authors declare that the research was conducted in the absence of any commercial or financial relationships that could be construed as a potential conflict of interest.
